# Higher Education Institutions: An Underused Setting for Addressing Adverse Childhood Experiences

**DOI:** 10.5888/pcd23.260495

**Published:** 2026-10-29

**Authors:** Sarah Zhang, Akilah Collins-Anderson

**Affiliations:** 1 Wayne State University, Detroit, Michigan

Adverse childhood experiences (ACEs) are potentially traumatic experiences that occur from birth through age 17 years and can undermine a child’s safety and stability (1). From various forms of abuse and neglect to other aspects that can impair a child’s well-being, ACEs are a major public health concern. In 2024, the National Survey of Children’s Health found that approximately 38% of children experienced at least 1 ACE (2). Among US adults, 64% reported at least 1 ACE, and nearly 18% have reported 4 or more (3). These experiences warrant attention because the effects may accumulate, potentially resulting in lifelong adverse health and social consequences.

People exposed to ACEs are more likely to report mental illness (4), substance use (5), interpersonal violence (6), and other health-risk behaviors. The cumulative effects of ACEs may also contribute to toxic stress, disrupt healthy development, and increase vulnerability to chronic health conditions later in life (7). Because these health outcomes and potential negative effects occur across the life course, efforts to reduce exposure to ACEs are critical.

Preventing ACEs requires creating safe, stable, and nurturing relationships and environments that reduce children’s exposure to adversity (8). However, opportunities to identify and respond to ACEs through screening, referral, and supportive services may not be accessible to all. These challenges warrant consideration of more opportunities to screen for ACEs and offer support beyond childhood for people with a history of ACEs. One promising approach is to focus on emerging adults. The objective of this essay is to describe how higher education institutions (HEIs) can serve as an opportune setting to address ACEs among emerging adults.

## Expanding the Focus on ACEs to Emerging Adulthood

Policies, programs, and early interventions can decrease the prevalence of ACEs by reducing children’s exposure to adversity and strengthen relationships and environments that protect their well-being. However, some approaches implemented during childhood rely on the involvement of parents or guardians, whose engagement may not always be available or possible. Extending attention beyond childhood provides opportunities to address earlier adversity as people enter new stages of life. Emerging adulthood, generally spanning ages 18 to 29 years, is a period during which people undergo transition, including navigating their independence, exploring their identity, and embracing their career and educational choices (9).

Focusing on emerging adults provides an opportunity to strengthen protective factors and, from a life course perspective, intervene before the consequences accumulate or become more pronounced (10). Access to supportive relationships, health and behavioral health services, stable housing and financial resources, and other forms of social support may help emerging adults navigate the effects of prior adversity while managing new challenges (11,12). In the early stages of this period, emerging adults may no longer be interacting with child- and family-serving systems through which ACE prevention and early intervention efforts are often delivered. Identifying settings that maintain sustained contact with emerging adults is therefore important for extending ACE-related support. For the millions of emerging adults who pursue postsecondary education, colleges and universities are one such setting.

## Strengths of HEIs in Addressing ACEs

Millions of emerging adults are enrolled in colleges and universities in the US, positioning HEIs to offer ACE-related support. Some HEIs have implemented efforts to address trauma and childhood adversity, such as The Ohio State University’s Advocacy and Resources for Trauma initiative (13); however, the potential role of HEIs in addressing ACEs extends beyond individual programs or initiatives. Recognition of the opportunity for HEIs to assume a greater role in addressing trauma and adversity is growing (14,15).

As complex institutions, HEIs engage emerging adults across multiple aspects of their lives and have many strengths that make them an ideal setting: for example, their infrastructure, resources, established systems of support, and opportunities for social support. Thus, a university-wide approach that includes student screenings, referrals, and trauma-informed interventions can be advantageous. The diverse resources, relationships, and organizational structures found at HEIs provide a strong foundation and capacity to promote student health and play a meaningful role in addressing ACEs. For this essay, we outline 2 examples of strengths, existing infrastructure and the social environment.

Established infrastructure is one of the greatest strengths of HEIs. Their institutional capacity allows ACE-related efforts to be integrated into existing systems and, where feasible, coordinated across multiple areas of the institution. For example, communication platforms (eg, Canvas, social media) are routinely used to announce campus events, share resources, and connect with students. These platforms could similarly be used to disseminate key information about ACEs, increase awareness of available resources, and highlight available campus- or community-based services. HEIs also have varying levels of a health infrastructure to support student well-being, ranging from wellness and counseling centers to health clinics, pharmacies, and at some institutions, university-affiliated hospitals. Clinics and counseling centers may incorporate trauma-informed practices, identify students who may benefit from additional support, and establish referral pathways. Moreover, because HEIs are valuable sources of health care and supportive services, particularly for students who have relocated to attend college, these services and pharmacies can support continuity of care for those receiving treatment related to past trauma or adversity (16).

Another strength is the social environment. Through residential life, extracurricular activities, campus events, and other opportunities for collegiate interaction, students are in an environment that can help them develop their social network and support systems. The many student organizations and groups on campus, for example, can offer a sense of belonging and community, an essential protective factor (17). Supportive relationships with faculty, advisors, and other trusted adults may also serve as a protective factor for students who have a history of trauma or abuse, potentially serving as a vital resource that fosters resilience and emotional safety (18). As students’ needs and circumstances evolve throughout their educational journey, positive interactions with others may increase their willingness to participate in ACE-related initiatives or support their healing journey. Ultimately, the social aspect of attending an HEI is a critical strength. Beyond the classroom, campuses support positive student interactions and provide opportunities for students to build meaningful relationships that can provide sources of social and emotional support for students.

## Proposed Solution

HEIs could use a multidisciplinary approach in their existing systems to address ACEs. Having an all-hands-on deck approach can also reduce pressure on counseling offices to a more holistic system — one that includes faculty, staff, and students. We propose a comprehensive, institution-wide approach in which the university ecosystem communicates and collaborates (Figure).

**Figure:**
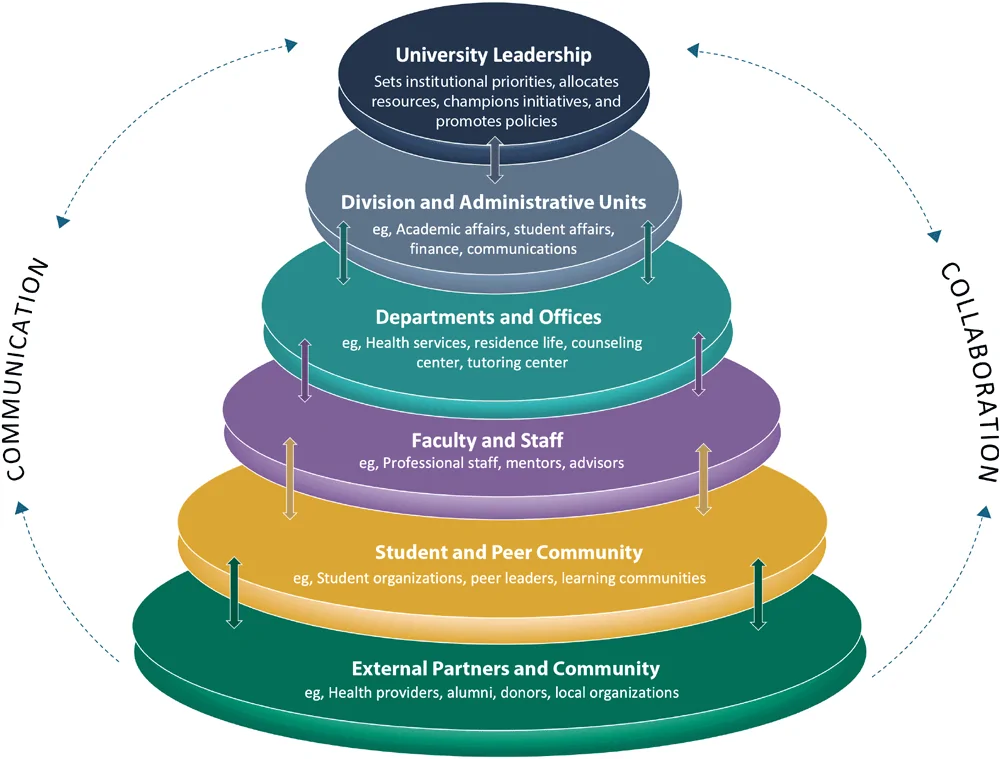
Multilevel university framework for addressing adverse childhood experiences.

University leadership can set institutional priorities, allocate resources, champion initiatives, and promote policies that support ACE-related initiatives. Commitment from leadership can help ensure that ACE-related efforts are strategically embedded, establishing potential long-term sustainability and coordinated support. Leaders can work with all levels of the university, including divisions, administration units, departments, and offices to offer trauma and adversity awareness seminars or support faculty training to incorporate trauma-informed teaching. They can also support residence life departments in offering optional, confidential ACE screenings for all students.

Routine screenings are underused despite growing recognition of the lasting effects of ACEs (19). Although some people may not be aware of the benefits of screening, ACE screening may help the university support students at increased risk for behavioral health concerns, chronic disease, academic difficulties, and other adverse outcomes. Screening may also facilitate timely referrals and connect students with services. Health services can coordinate with counseling centers and other campus services to ensure that students have a direct referral and support pathway. Moreover, student affairs offices can offer ACE-focused workshops to alleviate stigma and increase awareness about ACEs.

Faculty and staff are essential partners because they often serve as the first point of contact for students experiencing academic, behavioral, or emotional difficulties. Training can help them recognize signs of distress and refer students to counseling and resources. In addition, if a student were to disclose an ACE, instructors could be trained on how to navigate the conversation without shame or assumptions. Mentorship programs are common in university communities, and establishing such a program for students who are navigating recently recognized trauma can help students. Creating peer mentorship programs in which students themselves are trauma-informed mentors can help ease the burden on strained counseling services and can also help build healthy social connections.

Student organization leaders could also be trained to recognize signs of distress among the student body and create a streamlined process of communication between club board members and supportive services. Hosting campus-wide events, such as wellness walks and social gatherings, can bring students together and can help reduce toxic stress. Promoting these organizations and events requires cross-communication and collaboration so that students are aware of the available opportunities for connection and support.

Institutions can further strengthen ACE identification and interventions through external partnerships with local organizations and the community. Behavioral health providers, public health agencies, community organizations, and social service agencies can expand access to specialized services that may not be available on campus. These partnerships can facilitate referrals for trauma-focused treatment, substance use services, and other forms of social support.

## Implications for Public Health

An interdisciplinary approach that engages multiple areas of a university may encourage entire divisions of university systems to work together and offer a promising strategy to address ACEs. By leveraging existing infrastructure, services, and relationships across campus, HEIs have an opportunity to develop more coordinated approaches for ACE identification, referral, and support. If these efforts are integrated and championed across levels, from university leadership to external partners and organizations, students are likely to benefit. Such efforts can support academic success, mental health, social relationships, and resilience. Addressing ACEs should not be the responsibility of a single department or office when multiple systems in the institution can be leveraged through coordinated efforts.

Additional research is needed to determine the feasibility of designing, implementing, and sustaining an interdisciplinary and university-wide initiative. Feasibility studies could identify institutional resources, barriers, and facilitators. Future research should also include short- and long-term evaluations to determine whether coordination increases ACE screening and health-seeking behavior. Leveraging the existing strengths of HEIs can be a remarkable opportunity to introduce and extend ACE-related support for emerging adults as they navigate this stage of their life course.
